# Medication Optimization Among People With Type 2 Diabetes Participating in a Continuous Glucose Monitoring–Driven Virtual Care Program: Prospective Study

**DOI:** 10.2196/31629

**Published:** 2022-04-05

**Authors:** Amit R Majithia, David M Erani, Coco M Kusiak, Jennifer E Layne, Amy Armento Lee, Francis R Colangelo, Robert J Romanelli, Scott Robertson, Shayla M Brown, Ronald F Dixon, Howard Zisser

**Affiliations:** 1 Department of Medicine University of California San Diego School of Medicine La Jolla, CA United States; 2 Department of Pediatrics University of California San Diego School of Medicine La Jolla, CA United States; 3 Onduo Professionals PC Newton, MA United States; 4 Verily Life Sciences South San Francisco, CA United States; 5 Onduo LLC Newton, MA United States; 6 Allegheny Health Network Monroeville, PA United States; 7 Sutter Health Palo Alto, CA United States

**Keywords:** continuous glucose monitoring, digital health, GLP-1 receptor agonist, HbA1c, telemedicine, type 2 diabetes, monitoring, diabetes, optimization, medication, virtual care, prospective, app, lifestyle, coaching, self-management

## Abstract

**Background:**

The Onduo virtual care program for people with type 2 diabetes (T2D) includes a mobile app, remote lifestyle coaching, connected devices, and telemedicine consultations with endocrinologists for medication management and prescription of real-time continuous glucose monitoring (RT-CGM) devices. In a previously described 4-month prospective study of this program, adults with T2D and baseline glycated hemoglobin (HbA_1c_) ≥8.0% to ≤12.0% experienced a mean HbA_1c_ decrease of 1.6% with no significant increase in hypoglycemia.

**Objective:**

The objective of this analysis was to evaluate medication optimization and management in the 4-month prospective T2D study.

**Methods:**

Study participants received at least 1 telemedicine consultation with an Onduo endocrinologist for diabetes medication management and used RT-CGM intermittently to guide therapy and dosing. Medication changes were analyzed.

**Results:**

Of 55 participants, 48 (87%) had a medication change consisting of a dose change, addition, or discontinuation. Of these, 15 (31%) participants had a net increase in number of diabetes medication classes from baseline. Mean time to first medication change for these participants was 36 days. The percentage of participants taking a glucagon-like peptide-1 receptor agonist increased from 25% (12/48) to 56% (n=27), while the percentages of participants taking a sulfonylurea or dipeptidyl peptidase 4 inhibitor decreased from 56% (n=27) to 33% (n=16) and 17% (n=8) to 6% (n=3), respectively. Prescriptions of other antidiabetic medication classes including insulin did not change significantly.

**Conclusions:**

The Onduo virtual care program can play an important role in providing timely access to guideline-based diabetes management medications and technologies for people with T2D.

**Trial Registration:**

ClinicalTrials.gov NCT03865381; https://clinicaltrials.gov/ct2/show/NCT03865381

## Introduction

### Background

The Centers for Disease Control and Prevention (CDC) estimates that approximately 35 million people have type 2 diabetes (T2D), which is approximately 11% of the US population [1]. The incidence and prevalence of T2D continue to climb and outcomes are not improving despite increases in the number of effective drugs and advances in management technology. A growing body of evidence suggests that telehealth programs for T2D including smartphone apps, connected devices, and remote lifestyle coaching may help to address this public health crisis [2]. Participation in these programs has been associated with improvement in glycated hemoglobin (HbA_1c_) and other related comorbidities [3-14]. Recently, telehealth programs have begun to incorporate medical management and the use of advanced remote monitoring technology; however, data on methodologies and outcomes are limited [13,15].

The Onduo virtual care program for adults with T2D provides access to video consultations with endocrinologists for medication management and prescription of real-time continuous glucose monitoring (RT-CGM) devices, which are used to facilitate optimization of medication regimens and lifestyle coaching and participant self-management. A recent prospective trial of the Onduo program reported a reduction in laboratory-measured HbA_1c_ of 1.6% (SD 1.0; *P*<.001) from 8.9% (SD 1.0) to 7.3% (SD 0.9), and a significant improvement in continuous glucose monitoring (CGM)–derived glycemic metrics at 4 months in 55 adults with suboptimally controlled T2D [13]. Here we report the detailed medication management that occurred during the study.

### Study Objective

The objective of this analysis was to evaluate remote medication management by Onduo endocrinologists during telemedicine visits in conjunction with RT-CGM use among participants who completed the recent 4-month prospective trial [13].

## Methods

### Participants

Detailed study methods have been previously reported [13]. In brief, participants were ≥18 years of age, had a confirmed diagnosis of T2D, had HbA_1c_ level ≥8.0% and ≤12.0%, were willing to use a blood glucose monitor and CGM device, and owned a smartphone. Major exclusion criteria included malignant cancer, dialysis or end-stage renal disease or dialysis, liver or pancreatic failure, cystic fibrosis, chronic heart failure, or use of an insulin pump.

### Protocol

Baseline and final assessments were conducted in person at the 2 study sites and included physical measures, blood draws, and questionnaires. The intervention was conducted remotely through the Onduo virtual care program.

### Virtual Care Program Participation

The Onduo virtual care program for adults with T2D combines connected devices, remote lifestyle coaching, and clinical support with a mobile app has been previously described [11,12,14]. In this study, participants were asked to engage at least once per week with their health coach or care team and to participate in a telemedicine consultation with an Onduo endocrinologist. All participants were mailed a RT-CGM device (Dexcom G6, Dexcom) for intermittent use as indicated in the American Diabetes Association (ADA) Standards of Medical Care in Diabetes for adults with T2D [16]. Participants were asked to wear six 10-day sensors, with an initial period of 20 days (2 sensors); the remaining 4 sensors were worn on a 10-day “on” and 11-day “off” cycle. CGM glucose data were used by the care team for education and lifestyle coaching on the impact of diet and exercise and by the Onduo endocrinologists for medication management and to evaluate the efficacy of medication changes on glycemic control. Optimization of medication regimens was done remotely by endocrinologists and incorporated medical history, laboratory test results, and patient preferences, and was done in accordance with the ADA Standards, which is analogous to an in-person clinical practice [16]. The ADA Standards indicate that glucagon-like peptide-1 (GLP-1) receptor agonists are recommended as the first injectable medication, preferable to insulin. GLP-1 receptor agonists are also preferred over dipeptidyl peptidase 4 (DPP-4) inhibitors and sulfonylureas due to greater potency and positive cardiovascular outcomes. In addition, GLP-1 receptor agonists have superior side effect profiles compared to sulfonylureas, which carry risks of severe hypoglycemia and weight gain [17].

### Statistical Analysis

Change from baseline at 4 months for clinical characteristics and the number of diabetes medication classes prescribed to participants were evaluated by Wilcoxon signed-rank tests. The change in number of participants prescribed specific diabetes medication classes was evaluated by McNemar exact tests. Statistical significance was defined as *P*<.05. All analyses were performed in Python 3.6.7 (Python Software Foundation).

### Ethics

The study protocol and informed consent forms were approved by the Western Institutional Review Board (20182873) and registered with ClinicalTrials.gov (NCT03865381).

## Results

### Participants

Of 55 participants, 48 (87%) had a medication change. Baseline and follow-up demographic and clinical characteristics of the cohort with a medication change are presented in Table 1. At baseline, 8% (4/48), 44% (n=21), and 48% (n=23) of participants were prescribed 1, 2, and ≥3 classes of diabetes medications, respectively.

**Table 1:** Participant baseline and follow-up characteristics (N=48).

Parameter		Baseline	Follow-up	*P* value	
**Gender**					N/A^a^
	Male, n (%)	26 (54)	26 (54)		
	Female, n (%)	22 (46)	22 (46)		
Age (years), mean (SD)		56.9 (11.1)	56.9 (11.1)	N/A	
Weight (pounds), mean (SD)^b^		217.74 (60.5)	208.7 (54.4)	<.001	
BMI, mean (SD)^b^		33.5 (7.0)	32.2 (6.3)	<.001	
Baseline HbA_1c_^c^ (%), mean (SD)		8.9 (1.0)	7.3 (1.0)	<.001	
**Number of diabetes medication classes, n (%)**					.03
	0	0 (0)	0 (0)		
	1	4 (8.3)	3 (6.2)		
	2	21 (43.8)	14 (29.2)		
	≥3	23 (47.9)	31 (64.6)		
Systolic blood pressure (mm Hg), mean (SD)^b^		133.6 (16.2)	128.4 (17.1)	.02	
Diastolic blood pressure (mm Hg), mean (SD)^b^		81.5 (10.7)	80.5 (10.9)	.46	
Total cholesterol (mg/dL), mean (SD)		169.9 (42.6)	151.8 (42.1)	<.001	
HDL^d^ cholesterol (mg/dL), mean (SD)		40.2 (9.6)	39.0 (11.0)	.35	
LDL^e^ cholesterol (mg/dL), mean (SD)		101.8 (36.1)	94.4 (32.3)	.01	
Non-HDL cholesterol (mg/dL), mean (SD)		129.8 (42.6)	112.7 (38.7)	<.001	
Total cholesterol/HDL ratio, mean (SD)		4.5 (1.4)	4.0 (1.3)	.006	
Triglycerides (mg/dL), mean (SD)		242.7 (205.1)	197.0 (172.0)	.007	

^a^N/A: not applicable.

^b^As one subject did not complete the 4-month assessment at the study site, but submitted results from an external laboratory, n=47.

^c^HbA_1c_: glycated hemoglobin.

^d^HDL: high-density lipoprotein.

^e^LDL: low-density lipoprotein.

### Medication Management

The baseline and final medications at 4 months are presented in Table 1 and Table 2. Time to first medication change was 36.4 (SD 17.6) days. The most notable changes were a decrease in use of DPP-4 inhibitors, a decrease in sulfonylurea use, and an increase in GLP-1 receptor agonist use from baseline to 4 months. Medication changes including additions, discontinuations, and dose changes are presented in Table 2. Overall, 31.3% (15/48) of participants had an increase in the number of classes of diabetes medications prescribed from baseline and 10.4% (n=5) of participants had a decrease in number of classes of diabetes medications from baseline. In addition, 31.3% (n=15) of participants had a dose titration of an existing medication or a new medication.

**Table 2:** Participant baseline and follow-up medications by drug class (N=48).

Diabetes medication classes	Participants, n (%)			
	Baseline	Baseline medication stopped	Medication addition during study	Follow-up
Alpha glucosidase inhibitor	1 (2.1)	0 (0)	0 (0)	1 (2.1)
Biguanide	40 (83.3)	0 (0)	3 (6.3)	43 (89.6)
DPP-4^a^ inhibitor	8 (16.7)	7 (14.6)	2 (4.2)	3 (6.2)
GLP-1^b^ receptor agonist	12 (25)	1 (2.1)	16 (33.3)	27 (56.2)
Insulin	18 (37.5)	0 (0)	2 (4.2)	20 (41.7)
SGLT2^c^ inhibitor	18 (37.5)	2 (4.2)	3 (6.3)	19 (39.6)
Sulfonylurea	27 (56.2)	13 (27.1)	2 (4.2)	16 (33.3)
Thiazolidinedione	1 (2.1)	0 (0)	0 (0)	1 (2.1)
Other diabetes medications	0 (0)	0 (0)	4 (8.3)	4 (8.3)

^a^DPP-4: dipeptidyl peptidase-4.

^b^GLP-1: glucagon-like peptidase-1.

^c^SGLT2: sodium/glucose cotransporter 2.

**Figure 1:**
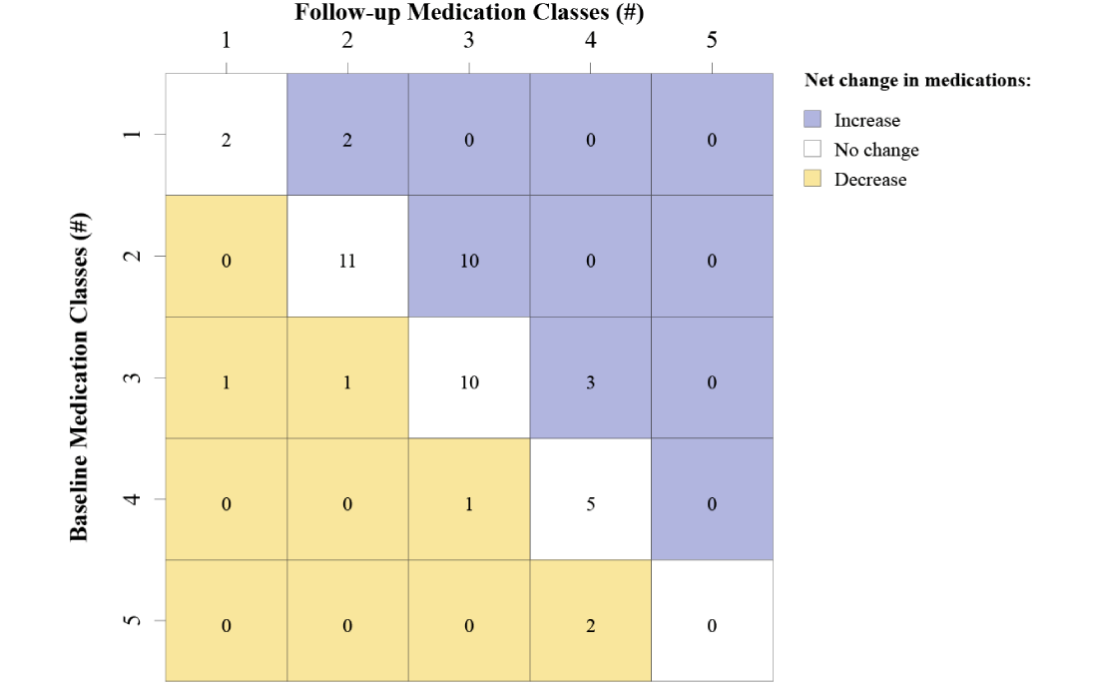
Number of participants and net change in number of medication classes prescribed from baseline to follow-up. The value in each cell represents the number of participants with a net increase, net decrease, or no change in number of medication classes prescribed from baseline to follow-up.

At a more granular level, the pattern of medication changes consisting of additions and discontinuations varied according to the baseline number of medication classes prescribed (Figure 1). The value in each cell represents the number of participants with a net increase, net decrease, or no change in number of medication classes prescribed from baseline to follow-up. Addition of a class was most frequently observed (n=10) for individuals on 2 classes at baseline. No additional classes were prescribed for individuals on 4 or 5 medications at baseline. Discontinuations were observed for individuals on 3, 4, or 5 medications at baseline and in one instance an individual on 3 classes at baseline had a final regimen of only 1 class at the end of the study.

### Change in HbA_1c_

HbA_1c_ for the present cohort (n=48) decreased by 1.6% (SD 1.0; *P*<.001) at 4 months, which was similar to change in HbA_1c_ reported for the full study cohort (n=55).

### Use of RT-CGM for Medication Management Case Study

**Figure 2:**
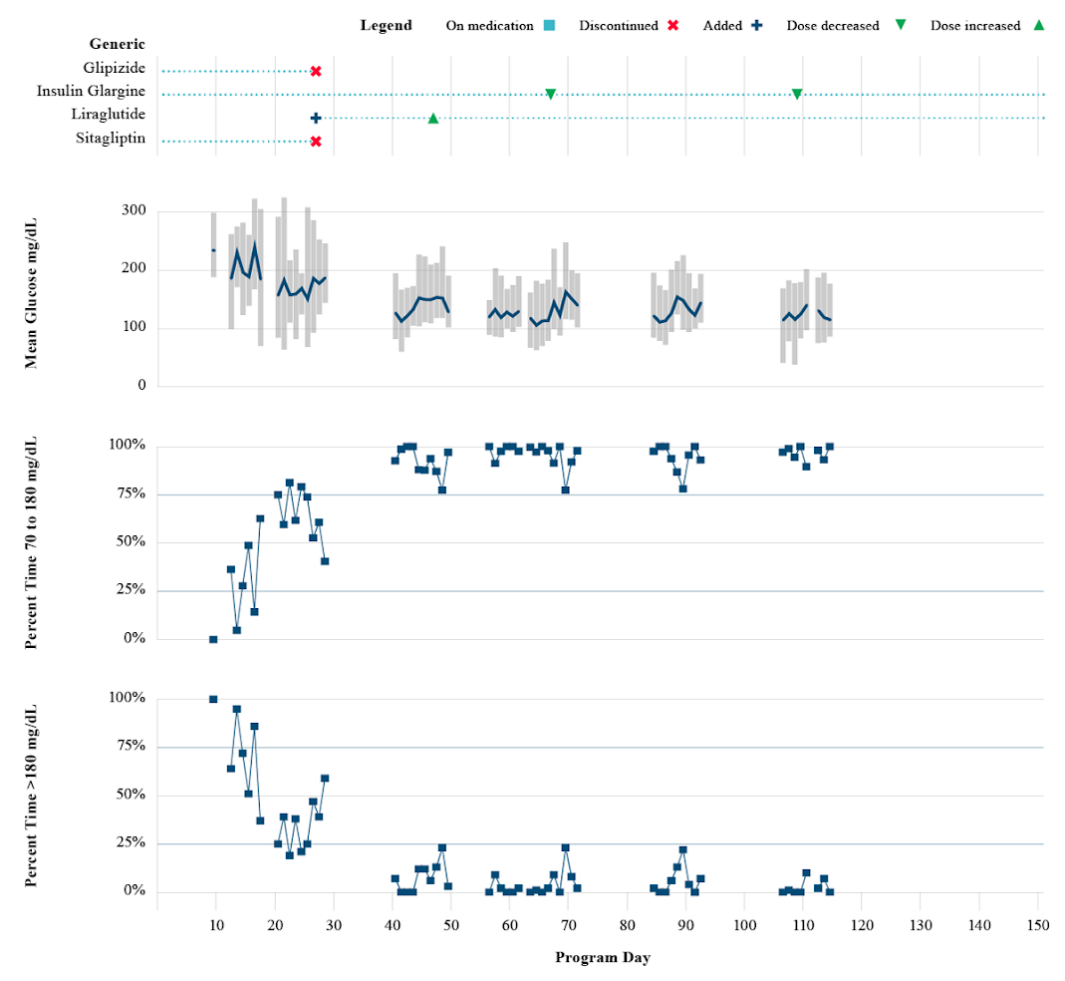
Medication changes over 4 months for one sample participant (use of real-time continuous glucose monitoring for medication management). A total of 4 medications changes were made: day 27, sulfonylurea was discontinued due to concurrent insulin use, and the DPP-4 inhibitor was discontinued and exchanged for a GLP-1 receptor agonist to address hyperglycemia; day 47, GLP-1 receptor agonist dose was increased due to glycemic excursions >200 mg/dL; day 67, basal insulin was decreased; day 108, insulin was further decreased due to small increases in percent time <70 mg/dL. DPP-4: dipeptidyl peptidase 4; GLP-1: glucagon-like peptide-1.

**Figure 3:**
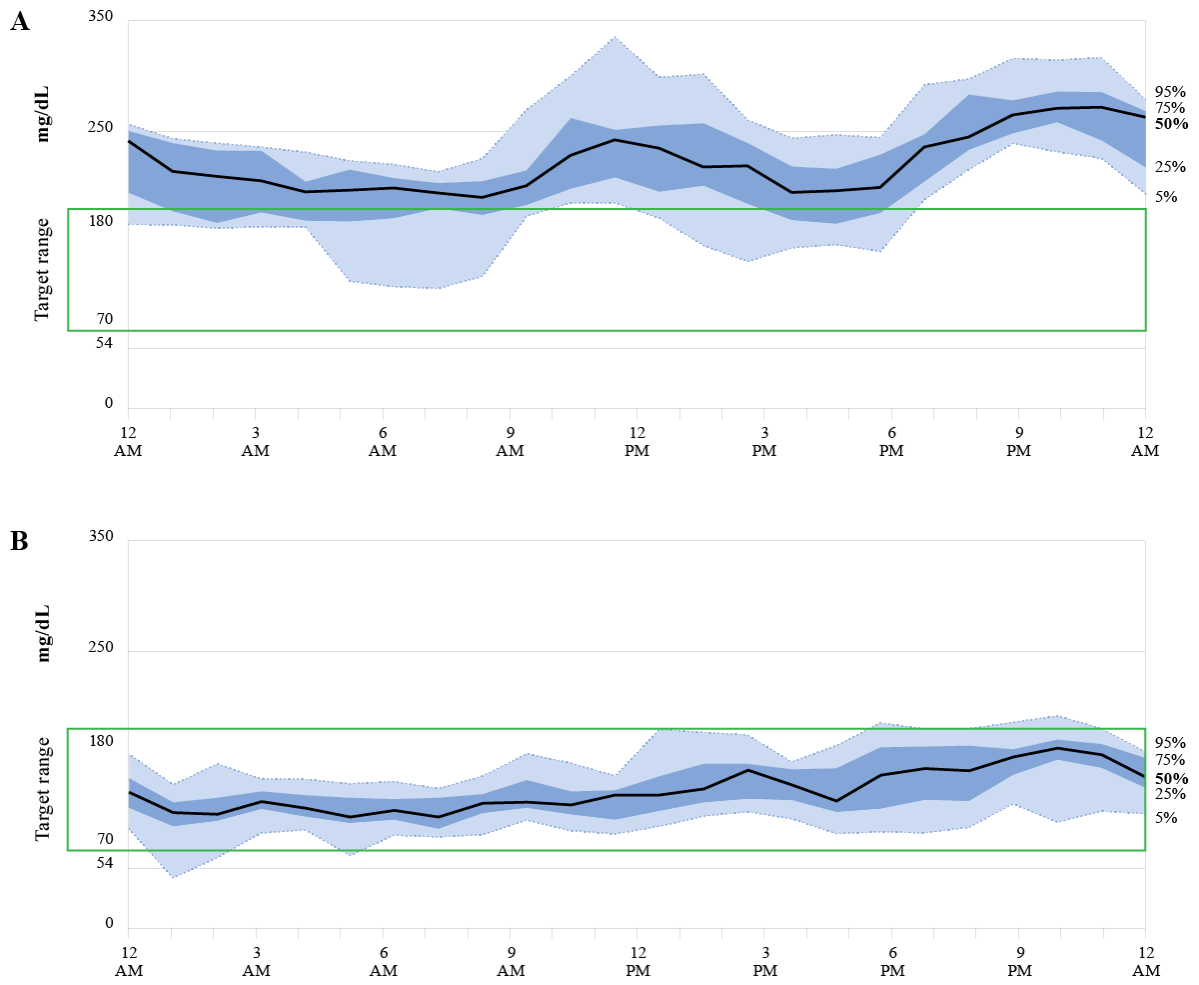
Median glycemic response over 24 hours for one sample participant. Comparison of the glycemic response for the (A) first 10-day real-time continuous glucose monitoring sensor wear period to the (B) last 10-day sensor wear period demonstrates less glycemic variability and reductions in postprandial glycemic excursions. Baseline HbA_1c_ improved from 12.0% to 6.3% at 4 months; baseline weight decreased 25 pounds at 4 months.

Figure 2 demonstrates the use of intermittent RT-CGM to accomplish medication management in a female study participant aged 66 years, baseline HbA_1c_ 12.0% and weight 174 pounds, taking 3 antidiabetes medications (glipizide, insulin glargine, and sitagliptin) upon entering the study. A total of 4 medications changes were made over the course of the 4-month study. Baseline sensor wear (two 10-day sensors) revealed mean glucose values ranging from 200-250 mg/dL and time in range (TIR) 70-180 mg/dL of <50%, with a few readings <70 mg/dL. Based on this glucose profile and the use of insulin, the Onduo endocrinologist made the following changes on day 27: the sulfonylurea (glipizide) was discontinued due to concurrent insulin use and the DPP-4 inhibitor (sitagliptin) was discontinued and exchanged for a GLP-1 receptor agonist (liraglutide) to address hyperglycemia. The dose of the GLP-1 receptor agonist was increased on day 47 due to glycemic excursions >200 mg/dL. Basal insulin was decreased on day 67. Further decreases in insulin were observed at day 108 in response to a small increase in CGM readings <70 mg/dL to minimize the risk of hypoglycemia. A comparison of the first CGM wear period to the last demonstrates less glycemic variability and reductions in postprandial glycemic excursions (Figure 3). The participant had a follow-up HbA_1c_ of 6.3% and lost 25 pounds during the 4-month study period.

## Discussion

### Principal Findings

This prospective study of adults with suboptimally controlled T2D participating in the Onduo virtual care program resulted in improved glycemic control, which was achieved through a unique model of telemedicine consultations with specialists for medication management combined with remote health coaching and access to mid-level providers, all facilitated by intermittent use of RT-CGM. The major patterns of medication changes were discontinuation of sulfonylureas in approximately half of participants and a 2-fold increase in the number of participants prescribed GLP-1 receptor agonists over the course of the 4-month study.

Clinical inertia is a key barrier to optimal diabetes management, prolonging poor glycemic control and, as a result, increasing the likelihood of diabetes complications [18]. The rapid therapeutic feedback loop enabled by the Onduo care model through timely sharing of glycemic data and patient-provider communication is likely a key factor in reducing time to therapeutic intensification/change. In clinical practice, the delay in therapeutic intensification is prolonged, and tends to occur at a higher rate among patients already taking ≥2 oral antidiabetic drugs [19], which was the case for 92% (44/48) of participants at baseline. A recent study reported that approximately 50% of patients with an HbA_1c_ level between 8.0% and 8.9% on 2 oral antidiabetic drugs had no therapy intensification for 6 months following the identification of poor glycemic control [20].

Optimizing medication regimens in line with evidence-based guidelines, as was done in this study, is essential to improving glycemic control. Although GLP-1 receptor agonists are recommended based on efficacy, better side effect profiles, and demonstrated cardiovascular benefit, some reports indicate that these are prescribed in <6% of people with T2D, even those with established cardiovascular disease [20,21]. In contrast, by the end of this study, 56% (26/48) of participants were prescribed a GLP-1 receptor agonist and only 2 participants had therapeutic intensification with insulin. Thus, the data from the present analysis, when taken in context of the clinically significant improvements in HbA_1c_ and TIR we previously reported [13], support early medication intensification and optimization in people taking multiple oral antidiabetic drugs who are particularly susceptible to clinical inertia [4]. The resulting improvement in glycemic control we observed, if sustained, may lower incidence of diabetes complications.

In addition to the Onduo program, there are several telehealth programs for T2D that have reported improvement in HbA_1c_ levels [3-10]. Although some of these programs are beginning to incorporate CGM use and telemedicine visits, to our knowledge there is only one recent study of a digital health program for people with T2D reporting medication management with CGM use [15]. However, the results of that study are not directly comparable to the present study due to substantial differences in study objectives and designs. This report by Shamanna et al [15] focused on deprescribing medications in participants provided a 90-day intensive nutrition intervention program in a retrospective design that analyzed only the 72% (64/89) of participants who were at least 60% adherent to the program.

The present study by contrast included the full range of medication management including class substitutions, dose titration, and additions, as well as deprescribing, in a prospective trial with an intent to treat analysis. Baseline glycemic control as measured by CGM was also better and met the clinical target [22] in participants in the Shamanna study (87% TIR versus 65% in this study). Both programs studied support the utility of CGM to improve short-term glycemic control, although longer duration studies are needed to evaluate acceptability, durability, and long-term clinical benefit for people with T2D.

Limitations of our study include the small sample size, the short duration, and lack of a randomized control arm, which limit the generalizability of our results. The intervention was intensive, which may not be reproducible or feasible for real-world telemedicine program participation. Although we were able to track and report recommended medication changes precisely in relation to real-time glycemia, we were not able to verify that prescriptions were filled from claims data due to the duration of the study. Thus, it is possible that participants did not make all the reported medication changes. In addition, we are not able to distinguish the clinical effects of individual components of the program (ie, lifestyle coaching, CGM use, and medication changes) from the program’s overall clinical efficacy.

### Conclusion

Although all aspects of the Onduo virtual care program may have contributed to improvement in glycemic control, telemedicine visits with endocrinologists and use of RT-CGM may have helped to overcome clinical inertia. Although further study is needed, optimization of medication regimens in line with current guidelines may be associated with long-term health benefits.

## Abbreviations

ADA: American Diabetes Association
CDC: Centers for Disease Control and Prevention
CGM: continuous glucose monitoring
DPP-4: dipeptidyl peptidase 4
GLP-1: glucagon-like peptide-1
HbA_1c_: glycated hemoglobin
RT-CGM: real-time continuous glucose monitoring
SGLT2: sodium/glucose cotransporter 2
T2D: type 2 diabetes
TIR: time in range

## Prior Presentation

Some of these data were presented at the 14th International Conference on Advanced Technologies & Treatments for Diabetes on June 2-5, 2021 [23].
